# Investigating the complex genetic architecture of ankle-brachial index, a measure of peripheral arterial disease, in non-Hispanic whites

**DOI:** 10.1186/1755-8794-1-16

**Published:** 2008-05-15

**Authors:** Sharon LR Kardia, M Todd Greene, Eric Boerwinkle, Stephen T Turner, Iftikhar J Kullo

**Affiliations:** 1 Department of Epidemiology, University of Michigan, Ann Arbor, Michigan 48109, USA; 2 Human Genetics Center and Institute of Molecular Medicine, University of Texas-Houston Health Science Center, Houston, Texas 77030, USA; 3 Division of Nephrology and Hypertension, and the Department of Internal Medicine, Mayo Clinic, Rochester, Minnesota 55905, USA; 4 Division of Cardiovascular Diseases, Department of Internal Medicine, Mayo Clinic, Rochester, Minnesota 55905, USA

## Abstract

**Background:**

Atherosclerotic peripheral arterial disease (PAD) affects 8–10 million people in the United States and is associated with a marked impairment in quality of life and an increased risk of cardiovascular events. Noninvasive assessment of PAD is performed by measuring the ankle-brachial index (ABI). Complex traits, such as ABI, are influenced by a large array of genetic and environmental factors and their interactions. We attempted to characterize the genetic architecture of ABI by examining the main and interactive effects of individual single nucleotide polymorphisms (SNPs) and conventional risk factors.

**Methods:**

We applied linear regression analysis to investigate the association of 435 SNPs in 112 positional and biological candidate genes with ABI and related physiological and biochemical traits in 1046 non-Hispanic white, hypertensive participants from the Genetic Epidemiology Network of Arteriopathy (GENOA) study. The main effects of each SNP, as well as SNP-covariate and SNP-SNP interactions, were assessed to investigate how they contribute to the inter-individual variation in ABI. Multivariable linear regression models were then used to assess the joint contributions of the top SNP associations and interactions to ABI after adjustment for covariates. We reduced the chance of false positives by 1) correcting for multiple testing using the false discovery rate, 2) internal replication, and 3) four-fold cross-validation.

**Results:**

When the results from these three procedures were combined, only two SNP main effects in *NOS3*, three SNP-covariate interactions (*ADRB2 *Gly 16 – lipoprotein(a) and *SLC4A5 *– diabetes interactions), and 25 SNP-SNP interactions (involving SNPs from 29 different genes) were significant, replicated, and cross-validated. Combining the top SNPs, risk factors, and their interactions into a model explained nearly 18% of variation in ABI in the sample. SNPs in six genes (*ADD2, ATP6V1B1, PRKAR2B, SLC17A2, SLC22A3, and TGFB3*) were also influencing triglycerides, C-reactive protein, homocysteine, and lipoprotein(a) levels.

**Conclusion:**

We found that candidate gene SNP main effects, SNP-covariate and SNP-SNP interactions contribute to the inter-individual variation in ABI, a marker of PAD. Our findings underscore the importance of conducting systematic investigations that consider context-dependent frameworks for developing a deeper understanding of the multidimensional genetic and environmental factors that contribute to complex diseases.

## Background

Atherosclerotic peripheral arterial disease (PAD) affects 8–10 million people in the United States [1,2] and is associated with a marked impairment in quality of life and an increased risk of stroke, myocardial infarction, and cardiovascular death [3]. Noninvasive assessment of PAD is performed by measuring the ankle-brachial index (ABI), the ratio of systolic blood pressure (SBP) at the ankle to the SBP in the arm. Normally ABI is ≥ 1.0, but with increasing narrowing of the lumen of arteries in the lower extremities, SBP at the ankle falls. Because individuals with PAD may not have typical symptoms of exertional leg discomfort, ABI values ≤ 0.95 or ≤ 0.90 have been used to diagnose the presence of PAD.

Coronary artery disease, cerebrovascular disease, and PAD are manifestations of the atherosclerotic disease process. As such, many of the well-established risk factors for atherosclerosis, such as increasing age, hyperlipidemia, hypertension, cigarette smoking and diabetes [4], contribute to these diseases. While these conventional risk factors have been associated with PAD, they explain < 20% of inter-individual variation in ABI [5]. The contribution of other 'novel' biochemical and genetic risk factors is less well characterized. In particular, little is known regarding genetic factors influencing inter-individual variation in ABI.

A recent review of the few association studies conducted to date suggests that the investigations of a small number of genes have failed to uncover compelling genetic determinants of PAD and most studies have only focused on the main effects of one polymorphism per gene [6]. We have previously reported findings from an association study that examined the relationships between variations in the *NOS3 *gene and ABI [7]. While this investigation also focused on a single gene, it extended the literature by employing a tag SNP approach to adequately cover variation and investigated the potential influence of 14 polymorphisms and related haplotypes on inter-individual ABI variation. Our findings provided evidence that *NOS3 *variants may have moderate effects on ABI variation, which is in line with the conventional wisdom that the effect of a single gene on a complex disease is expected to be modest and that genetic susceptibility to complex atherosclerotic disease is likely polygenic [6]. Furthermore, while the single candidate gene approach, largely employed to date, may offer valuable insights into the etiology of PAD, it fails to consider the interactive and context-dependent nature that defines complex diseases like PAD.

As a part of the Genetic Epidemiology Network of Arteriopathy (GENOA) study, genetic variants in a large collection of positional and biological candidate genes have been measured to better understand the contribution of genes to risk of arteriopathies that are associated with diseases of the heart, brain, kidneys, and peripheral arteries. Even with an increased understanding of the molecular genetic and biochemical basis of blood pressure (BP) regulation, lipoprotein metabolism, inflammation, oxidative stress, and glucose metabolism, it has been difficult to predict individual susceptibility to these diseases [8]. Complex traits, such as ABI, are influenced by a large array of genetic, environmental, behavioral, and social factors and their interactions [9]. As such, in order to develop a more complete picture of genetic susceptibility to PAD, it is necessary to move beyond the exclusive investigation of single gene effects. In this paper, we begin to characterize the complex genetic architecture of ABI by examining the effect of individual SNPs in candidate genes, interactions between SNPs and conventional risk factors, as well as interactions between SNPs within and across genes (intragenic and intergenic epistasis). In addition, we investigated whether the SNPs affecting ABI also influence 15 physiological and biochemical correlates of the pathways underlying variation in ABI. These include age, body mass index (BMI), smoking, SBP and diastolic blood pressure (DBP), fasting plasma cholesterol, high-density lipoprotein (HDL) cholesterol, triglycerides, C-reactive protein (CRP), homocysteine, lipoprotein (a) (Lp(a)), fibrinogen, hypertension, and diabetes. This paradigm shift to a more encompassing attempt to unravel the complex genetic architecture is an advance over the simplified single gene approach employed in the past. While difficult to dissect and interpret, a deeper understanding of interactive effects and underlying correlation structures will likely offer additional insights into the etiology of PAD and possible explanations for PAD susceptibility for certain individuals within particular contexts.

For this study, we identified 435 SNPs in 112 genes that have been previously implicated as playing a role in BP regulation, lipoprotein metabolism, inflammation, oxidative stress, and diabetes. To our knowledge, no other study has comprehensively investigated how this amount of variation in numerous candidate genes may influence PAD risk. A summary of the genes and their corresponding SNPs is provided [see Additional file 1]. Although association studies are favored over linkage studies for unraveling the genetic bases of complex disorders, lack of replication in a majority of such studies has been a major concern [10]. To reduce false positives we combined three approaches: adjustment for multiple testing using the false discovery rate (FDR) [11], internal replication, and cross-validation [12].

## Methods

### Study Population

Subjects included non-Hispanic white participants in the Genetic Epidemiology Network of Arteriopathy (GENOA) study, a community-based study of hypertensive sibships that aims to identify genes influencing BP [13,14]. The study was approved by the Institutional Review Board of Mayo Clinic, Rochester MN. Written informed consent was obtained from each participant. In the initial phase of the GENOA study (9/1995 to 6/2001), sibships containing ≥ 2 individuals with essential hypertension diagnosed before age 60 years were selected for participation. At the Rochester, MN field center, 1583 non-Hispanic whites were enrolled. Participants returned in Phase II of GENOA for physical examination, and measurement of non-conventional and novel risk factors as well as the ABI. Through November of 2004, ABI had been measured in 1046 participants.

### Clinical Assessments and Covariate Definitions

Height was measured by stadiometer, weight by electronic balance, and BMI was calculated as weight in kilograms divided by the square of height in meters. Resting SBP and DBP were measured by a random zero sphygmomanometer. Blood was drawn by venipuncture after an overnight fast. Serum total cholesterol and HDL cholesterol were measured by standard enzymatic methods. Low-density lipoprotein (LDL) cholesterol levels were calculated using the Friedewald formula [15]. The diagnosis of hypertension was established based on BP levels measured at the study visit (≥ 140/90 mmHg) or a prior diagnosis of hypertension and current treatment with antihypertensive medications. Diabetes was considered present if the subject was being treated with insulin or oral agents or had a fasting glucose level ≥ 126 mg/dL. Participants were considered as having "ever smoked" if they had smoked more than 100 cigarettes during their lifetime. CRP was measured by a highly sensitive immunoturbidimetric assay [16]. Fibrinogen was measured by the Clauss (clotting time based) method [17]. Lp(a) in serum was measured by an immunoturbidimetric assay using the SPQ™ Test System (Diasorin, Stillwater MN) as previously described [18]. Plasma homocysteine was measured by high-pressure liquid chromatography. Inter-assay coefficients of variance were: CRP, 2.6–2.8%; fibrinogen, 5.8–6.8%; Lp(a), 8.6–13.5%; homocysteine, 5.7–7.4%.

### Ankle-brachial index

ABI was measured in the supine position following a 5-min rest. Appropriately sized BP cuffs were placed on each arm and ankle, and a Doppler ultrasonic instrument (Medisonics, Minneapolis MN) was used to detect each pulse. The cuff was inflated to 10 mm Hg above SBP and deflated at 2 mm Hg/s. The first reappearance of the pulse was taken as the SBP. To calculate ABI, the SBP at each ankle site (posterior tibial and dorsalis pedis) was divided by the higher of the 2 brachial pressures. The lowest of the 4 ratios was designated as the ABI. The correlation of the lowest ABI with the average of the 2 ABIs from the same leg was 0.98, and inferences were similar using the lowest ABI or the average ABI.

### SNP Selection

Four hundred and thirty five SNPs from 112 genes known or hypothesized to be involved in BP regulation, lipoprotein metabolism, inflammation, oxidative stress, vascular wall biology, obesity and diabetes were identified from the genetic association literature and positional candidate gene studies [19]. These biological pathways and disease conditions are related to atherosclerosis. As PAD is an atherosclerotic process, studying variations in these candidate genes may yield insights into the genetic architecture of ABI. SNPs were chosen based on a number of different criteria including the published literature, non-synonymous SNPs with a minor allele frequency (MAF) > 0.02, and tag SNPs using public databases such as dbSNP [20] and Seattle SNPs [21].

Our algorithm for SNP selection first identified non-synonymous SNPs with a minor allele frequency (MAF) > 0.02 based on data from the Seattle SNPs database [21]. Second, we identified all SNPs with a MAF > 0.1 and unique sequence context that could potentially be typed in any of the three ethnic groups (non-Hispanic white, African-American, Hispanic) sampled in the GENOA study [13]. From the latter SNPs, tag SNPs were selected based on the *r*^2 ^method described by Carlson et al. [22]. The final list of SNPs to be genotyped was established by selecting 1 SNP from each bin pair according to the following selection prioritization: (first) a tag SNP in a conserved region (compared to mouse); (second) a tag SNP not in a conserved region; (third) a non-tag SNP in a conserved region; (fourth) neither a tag SNP nor a SNP in a conserved region. We used this priority system because several bins had multiple tag SNPs, and some bins had no identified tag SNPs.

### Genotyping

DNA was isolated using the PureGene DNA Isolation Kit from Gentra Systems (Minneapolis MN). Genotyping, based on polymerase chain reaction (PCR) amplification techniques, was conducted at the University of Texas-Health Sciences Center at Houston using the TaqMan assay and ABI Prism^® ^Sequence Detection System (Applied Biosystems, Foster City CA). Primers and probes are available from the authors upon request. Quality control measures for genotyping assays included robotic liquid handling; separate pre- and post-PCR areas; standard protocols and quality control analyses including 5% duplicates, positive and negative controls, computerized sample tracking, and data validity checks.

### Statistical Analysis

All analyses were carried out using the R statistical language [23]. Variables with skewed distributions were log transformed. Risk factor correlations were estimated using Pearson's product moment correlation. Allele and genotype frequencies were calculated using standard gene counting methods. Linkage disequilibrium (LD), as measured by r^2 ^[24], was estimated using an expectation maximization (EM) algorithm. Hardy-Weinberg Equilibrium was assessed using a chi-square test or Fisher's exact test if a genotype class had less than 5 individuals [25]. In all models, ABI was adjusted for age, sex, BMI, smoking status (ever vs. never), diabetes, and hypertension. Adjustment variables were chosen because they have known associations with PAD [2,26-29] or because they were statistically significant predictors of ABI in this dataset.

In the first stage of analysis, we tested for associations of each of the predictors (SNPs and demographic/biochemical risk factors) with ABI using least-squares linear regression methods [30]. We also tested for association between each single SNP and each risk factor to identify potential confounders. To determine whether interactions among predictors explained additional variation in ABI, we tested pairwise interactions among all possible pairs of predictors (i.e. SNP-SNP, SNP-risk factor, and risk factor-risk factor interactions). Associations involving interactions were assessed with a partial F test, which compares a full model that includes both the interaction terms and the main effects of the variables comprising the interaction terms to a reduced model that includes only the main effects.

To reduce false positives we used three different approaches: adjustment for multiple testing using FDR < 0.30 [11], internal replication with two subsets of unrelated individuals followed by testing for homogeneity of genotype-phenotype effects, and, finally, four-fold cross-validation (repeated 10 times) [31]. To create replication subsets, we randomly selected 1 hypertensive sib from each sibship without replacement to create Subset 1 and then randomly selected another hypertensive sib from each sibship to create Subset 2. The GENOA cohort contained a small number of singletons (i.e.- no matching sibs) that were equally divided between the two samples. A dichotomous "sample" variable was generated, with all subjects in Subset 1 assigned a value of 0 and all subjects in Subset 2 assigned a value of 1. If an effect was found to be significant in both subsets, modeling an interaction term between the significant SNP and the "sample" variable was used to assess the homogeneity of the respective genotype-phenotype effect. This interaction model was then compared to a reduced model without the "sample" interaction and significance was assessed with a partial F-test.

Cross-validation significantly reduces false positive results by eliminating associations that lack predictive ability in independent test samples. We performed four-fold cross-validation by dividing the full sample into four equally sized groups. Three of the four groups were combined into a training dataset, and the modeling strategy outlined above was carried out to estimate model coefficients. These coefficients were then applied to the fourth group, the testing dataset, to predict the value of the outcome variable of each individual in the independent test sample. This process was repeated for each of the 4 testing sets. Predicted values for all individuals in the test set were then subtracted from their observed values, yielding the total residual variability (SSE), $\sum_{i=1}^{n}{\left(y_{i}-{\hat{y}}_{i}\right)}^{2}$. The total variability in the outcome (SST) – the difference between each individual's observed value and the mean value for the outcome – was then calculated, $\sum_{i=1}^{n}{\left(\bar{y}-y_{i}\right)}^{2}$. In order to estimate the proportion of variation in the outcome predicted in the independent test samples, the cross-validated R^2 ^(CV R^2^) was calculated as follows: $CV\;R^{2}=\frac{SST-SSE}{SST}$. This cross-validation method provides a more accurate measure of the predictive ability of the genetic models and will be negative when the model's predictive ability is poor. Because random variations in the sampling of the four mutually exclusive test groups can potentially impact the estimates of CV R^2^, this procedure was repeated 10 times and the CV R^2 ^values were averaged [31]. Univariate associations were considered cross-validated if the average percent variation predicted in independent test samples was greater than 0.5% and interactions were considered cross-validated if the difference in average percent variation predicted in independent test samples between the full model containing the interaction term and the reduced model containing only main effect terms was greater than 0.5%.

To visualize the genetic architecture of ABI, we applied a novel data visualization scheme, the KGraph, described in Kelly et al. [32]. The KGraph was developed for the visualization of genetic association results and the underlying confounding due to SNP-SNP frequency correlations (i.e. LD), SNP-risk factor associations, and risk factor-risk factor correlations. It simultaneously displays both significant univariate associations and pairwise interactions with the outcome of interest, ABI, as well as the underlying correlation structure among the predictor variables (SNPs and risk factors).

Using a SNP list that was comprised of SNPs that passed our three filters (FDR, replication, and cross-validation), multivariable linear regression models combining the top SNPs, risk factors, and their interactions were then constructed and the percent variation in ABI explained by each model was estimated. Four-fold cross validation was used to estimate the predictive ability of these models in test samples not used to estimate the models.

## Results

The descriptive statistics for the full sample of non-Hispanic whites and the two subsets used to examine replication are presented in Table 1. The mean age was 59 years. The mean BMI was 31 kg/m^2^. The average ABI was 1.11. Fifteen percent of the participants had type II diabetes and 51% had a history of smoking.

**Table 1:** Descriptive Statistics for Study Participants

	**Full Sample (N = 1046)**	**Subset 1 (N = 330)**	**Subset 2 (N = 329)**	**Univariate association with ABI in full sample (N = 1046)**
	**Mean (± SD)**	**Mean (± SD)**	**Mean (± SD)**	**β estimate**

ABI	1.1 (0.1)	1.1 (0.2)	1.1 (0.1)	

Age, years	59.1 (10.1)	60.8 (9.1)	61.3 (9.0)	-3.6E-03***
BMI, kg/m^2^	30.9 (6.2)	31.1 (6.1)	31.9 (6.3)	2.9E-03***
Waist Hip Ratio, cm	0.91 (.11)	0.92 (0.1)	0.92 (0.1)	1.1E01*
SBP, mm Hg	131.1 (16.6)	135.1 (17.4)	135.0 (16.3)	-1.6E-03***
DBP, mm Hg	74.0 (9.0)	74.9 (9.7)	74.5 (9.2)	1.8E-03***
Pulse pressure, mm Hg	57.2 (15.3)	60.2 (15.8)	60.5 (16.1)	-2.5E-03***
Total cholesterol, mg/dL	196.7 (34.9)	193.0 (32.8)	195.2 (33.4)	-2.5E-05
HDL cholesterol, mg/dL	51.4 (14.9)	49.8 (14.3)	50.8 (14.0)	-8.1E-04**
LDL cholesterol, mg/dL	113.8 (31.8)	125.1 (36.5)	126.1 (36.6)	2.8E-04
Triglycerides, mg/dL	159.9 (96.1)	163.8 (97.7)	163.0 (90.0)	-7.4E-05
C-reactive protein, mg/L	0.45 (0.7)	0.50 (0.9)	0.45 (0.5)	-7.2E-03
Fibrinogen, mg/dL	318.3 (77.0)	321.0 (73.6)	324.7 (78.3)	-1.7E-04**
Homocysteine, umol/L	9.9 (2.7)	10.2 (2.8)	10.1 (2.8)	-4.9E-03**
Lp(a), mg/dL	40.1 (38.7)	40.5 (36.5)	40.4 (40.5)	-4.6E-04**

Female, n (%)	580 (55%)	170 (52%)	183 (56%)	-5.4E-02***
Ever smoker, n (%)	528 (51%)	174 (53%)	161 (49%)	-1.4E-02
Diabetes, n (%)	154 (15%)	48(15%)	71(22%)	-2.4E-02
Hypertensive, n (%)	773 (74%)	330 (100%)	329 (100%)	-3.7E-02***

*p < 0.05; ** p < 0.01; *** p < 0.001

ABI, ankle-brachial index; BMI, body mass index; SBP, systolic blood pressure; DBP, diastolic blood pressure; HDL, high density lipoprotein; LDL, low density lipoprotein; Lp(a), lipoprotein

In Table 2, we present a summary of the results from testing for SNP main effects, SNP-covariate, and SNP-SNP interactions and the number of associations that remained significant after adjustment for multiple testing (FDR < 0.3), testing for replication, and cross-validation. For example, 435 SNPs were evaluated for their association with adjusted ABI and 20 had FDR < 0.3, 3 internally replicated, and 5 cross-validated. Only two SNPs (located in the *NOS3 *gene, rs891512 and rs1808593) passed all three filters. In contrast, there were 6,926 tests of SNP-risk factor interactions and 20 had a FDR < 0.3, 72 internally replicated, but only 52 cross-validated. Only three SNP-risk factor interactions passed all three criteria – specifically, *ADRB2*_rs1042713 interacting with Lp(a) and *SLC4A5 *polymorphisms interacting with diabetes (Table 3). There were 91,113 tests of SNP-SNP interactions and we found 270 had a FDR < 0.3, 973 internally replicated, and 404 cross-validated. Only 25 SNP-SNP interactions passed all three criteria and are listed in Table 3.

**Table 2:** Quantitative summary of genetic associations with ABI that replicated, cross-validated, and passed FDR criterion

	**SNP Main Effects**	**SNP-Covariate Interactions**	**SNP-SNP Interactions**
Number of tests	435	6926	91113
P < 0.10 on full sample	77	815	10308
FDR (< 0.30) on full sample	20	20	270
Cross Validation (on full sample)	5	52	404
Replication (P < 0.10 in both groups)	3	72	973
FDR and Cross-validation	5	14	79
FDR and Replication	2	4	36
Replication and Cross-validation	2	7	52
FDR and Cross-Validation and Replication	2	3	25

FDR, False Discovery Rate

**Table 3:** Genetic effects that replicated, cross-validated, and passed FDR criterion

**Main Effects (2)**		**SNP**	**Subset 1 p-value**	**Subset 2 p-value**	**Full Sample p-value**	**R^2^**	**CV R^2^**
		*NOS3*_rs891512	0.0003	0.0458	0.0005	0.0151	0.0096
		*NOS3*_rs1808593	0.0006	0.0333	0.0001	0.0178	0.0105

**SNP-Covariate Interactions (3)**	SNP	Covariate				**R^2^**	**CV R^2^**

	*ADRB2*_rs1042713	Lp(a)	0.0847	0.0407	0.0006	0.0176	0.0089
	*SLC4A5*_rs828853	DIABETES	0.0564	0.0462	0.0004	0.0143	0.0095
	*SLC4A5*_rs12991424	DIABETES	0.0350	0.0278	0.0005	0.0106	0.0085

**SNP-SNP Interactions (25)**	**SNP1**	**SNP2**				**R^2^**	**CV R^2^**

	*TTRAP*_rs1129644	*HLA-DOA*_rs2581	0.0535	0.0608	0.0001	0.0198	0.0116
	*ADD2*_rs2110981	*TGFB3*_rs2268622	0.0355	0.0134	0.0004	0.0171	0.0115
	*ADD2*_rs2110981	*TGFB3*_rs2284791	0.0439	0.0049	0.0001	0.0200	0.0156
	*ADD2*_rs2270042	*AGT*_rs5049	0.0085	0.0765	0.0003	0.0171	0.0127
	*ADRB2*_rs1042713	*PRL*_rs1205960	0.0066	0.0885	0.0003	0.0175	0.0119
	*ATP6V1B1*_rs11681642	*IL1B*_rs3917356	0.0223	0.0105	0.0002	0.0195	0.0130
	*ATP6V1B1*_rs1024764	*ACCN4*_rs3755065	0.0395	0.0005	0.0002	0.0189	0.0152
	*ATP6V1B1*_rs2239484	*ACCN4*_rs3755065	0.0550	0.0005	0.0005	0.0161	0.0121
	*ATP6V1B1*_rs2239487	*ACCN4*_rs3755065	0.0238	0.0013	0.0004	0.0175	0.0123
	*AUP1*_rs10779958	*SLC2A2*_rs5400	0.0041	0.0868	0.0003	0.0155	0.0149
	*SCN7A*_rs1406275	*TGFB3*_rs3917195	0.0283	0.0004	0.0001	0.0180	0.0137
	*FGB*_rs4220	*KCNE4*_rs3795884	8.98E-05	0.0584	3.45E-05	0.0208	0.0163
	*GPC6*_rs1924115	*IL6*_rs2069827	0.0203	0.0338	0.0007	0.0145	0.0124
	*ICAM1*_rs5030352	*SLC19A3*_rs12185721	0.0869	0.0028	0.0001	0.0172	0.0073
	*MMP3*_rs683878	*VCAM1*_rs1041163	0.0644	0.0407	0.0003	0.0172	0.0085
	*MMP9*_rs20544	*SLC12A3*_rs2304483	0.0157	0.0876	0.0002	0.0180	0.0132
	*SLC4A5*_rs702462	*TGFB3*_rs3917187	0.0734	0.0010	0.0004	0.0176	0.0088
	*SLC4A5*_rs702462	*TGFB3*_rs3917201	0.0456	0.0010	7.65E-05	0.0207	0.0147
	*SLC4A5*_rs702462	*TGFB3*_rs3917210	0.0329	0.0009	4.62E-05	0.0222	0.0156
	*SLC4A5*_rs702462	*TGFB3*_rs3917211	0.0358	0.0015	5.91E-05	0.0212	0.0097
	*PRKAR2B*_rs257376	*SLC17A2*_rs1540273	0.0075	0.0036	0.0005	0.0174	0.0134
	*PRKAR2B*_rs257376	*SLC17A2*_rs2071299	0.0161	0.0020	0.0002	0.0182	0.0103
	*PLIN*_rs1052700	*TGFB3*_rs3917195	0.0126	0.0015	0.0006	0.0146	0.0054
	*SELE*_rs5368	*SLC22A3*_rs668871	0.0004	0.0954	0.0005	0.0167	0.0127
	*SELE*_rs5356	*SLC22A3*_rs668871	0.0009	0.0932	0.0006	0.0157	0.0124

All associations adjusted for age, gender, body mass index, smoking, diabetes, and hypertension.

*ACCN4 *amiloride-sensitive cation channel 4, pituitary; *ADD2 *adducin 2 (beta); ADRB2 *b2-adrenergic receptor; *AGT *angiotensinogen (serine (or cysteine) proteinase inhibitor, clade A (alpha-1 antiproteinase, antitrypsin), member 8); *ATP6V1B1 *ATPase, H+ transporting, lysosomal 56/58 kDa, V1 subunit B, isoform 1 (Renal tubular acidosis with deafness); *AUP1 *ancient ubiquitous protein 1; *FGB *fibrinogen, B beta polypeptide; *GPC6 *glypican 6; *HLA-DOA *human leukocyte antigen; *ICAM1 *intercellular adhesion molecule 1 (CD54), human rhinovirus receptor; *ILB1 *interleukin 1, beta; *IL6 *interleukin 6 (interferon, beta 2); *KCNE4 *potassium voltage-gated channel, Isk-related family, member 4; *MMP3 *matrix metalloproteinase 3 (stromelysin 1, progelatinase); *MMP9 *matrix metalloproteinase 9 (gelatinase B, 92 kDa gelatinase, 92 kDa type IV collagenase); *NOS3 *nitric oxide synthase 3 (endothelial cell); *PLIN *perilipin; *PRKAR2B *protein kinase, cAMP-dependent, regulatory, type II, beta; *PRL *prolactin; *SCN7A *sodium channel, voltage-gated, type VII, alpha; *SELE *selectin E (endothelial adhesion molecule 1); *SLC2A2 *solute carrier family 2 (facilitated glucose transporter), member 2; *SLC2A5 *solute carrier family 4, sodium bicarbonate cotransporter, member 5; *SLC12A3 *solute carrier family 12 (sodium/chloride transporters), member 3; *SLC17A2 *solute carrier family 17 (sodium phosphate), member 2; *SLC19A3 *solute carrier family 19, member 3; *SLC22A3 *solute carrier family 22 (extraneuronal monoamine transporter), member 3; *TGFB3 *transforming growth factor, beta 3; *TTRAP *TRAF and TNF receptor associated protein; *VCAM1 *vascular cell adhesion molecule 1.

**Figure 1:**
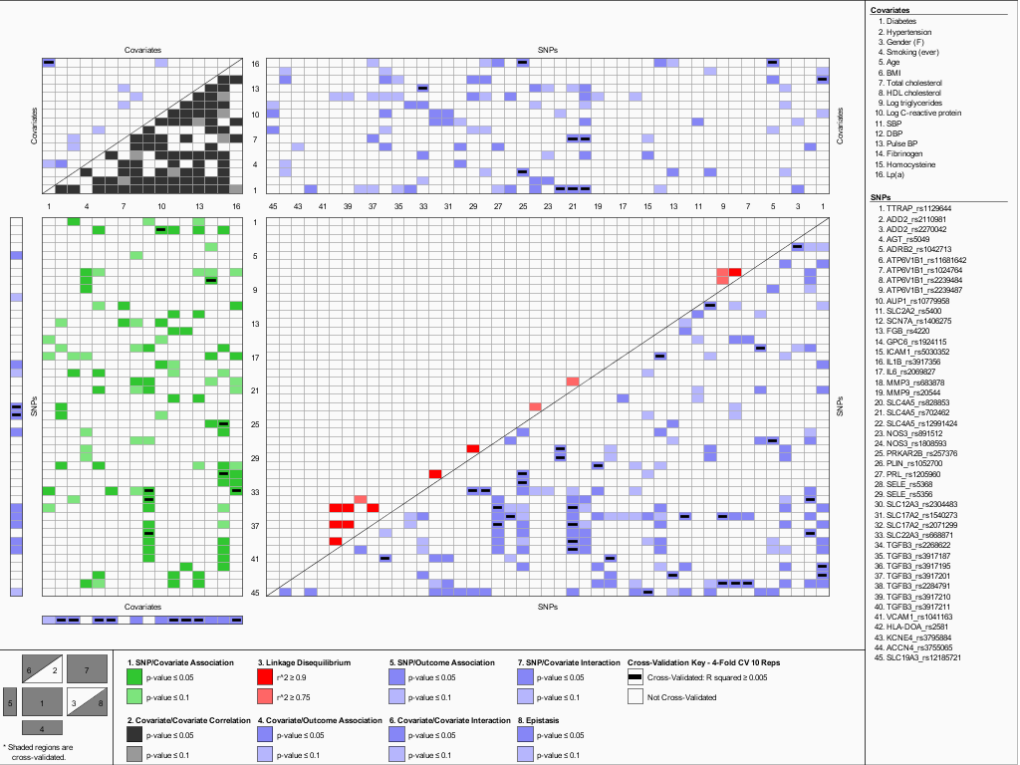
Genetic architecture of the ankle-brachial index in non-Hispanic Whites.

Figure 1 is a visual representation of the complex genetic and demographic/biochemical risk factor associations underlying variation in ABI. Using both color and spatial relationships, the KGraph presents both associations with ABI and the correlation structure of the predictors that underlie those associations. Only SNPs that passed all three filters are displayed, though all SNP-ABI, SNP-SNP (i.e. LD), and SNP-risk factor associations are represented to more fully understand the complex correlation structure underlying ABI predictors. Region 1, shown in green, displays the association between the SNPs and biochemical risk factors, one source of often overlooked confounding and information about underlying metabolic pathways. In this region, the cross-validated SNP associations with log triglyceride (*TGFB3 *and *SLC22A3*), log CRP (*ADD2*), fibrinogen (*ATP6B1*), homocysteine (*SLC17A2 *and *PKRAR2B*), and Lp(a) (*SLC22A3*) are indicated. Region 2, shown in grey, illustrates the correlations between the risk factors. The majority of the risk factors are significantly correlated (|r| < 0.3), with only Lp(a) levels not being highly correlated with other risk factors. The observed LD, shown in red in Region 3, occurs between SNPs that are within the same gene, with SNPs in the *TGFB3*, *SELE, NOS3*, and *SLC4A5 *genes being highly correlated.

The remaining regions are colored blue, indicating that they represent associations with ABI. Region 4, which displays the univariate association between risk factors and ABI shows that age, BMI, gender, hypertension, SBP, DBP, pulse pressure, and Lp(a) each have statistically significant and cross-validated associations with ABI. Region 5, which illustrates univariate associations between the SNPs and ABI, reveals that only two SNPs in *NOS3 *(which are in LD) have significant, replicated and cross-validated associations. Region 6 displays the risk factor-risk factor interactions significantly associated with ABI. Cross-validated interactions were observed between diabetes status and Lp(a). Region 7 displays the interactions between the SNPs and risk factors that were associated with ABI. Overall, we detected 10 statistically significant interactions between a variety of risk factors and SNPs that replicated and cross-validated. Upon controlling for multiple testing with FDR, only 3 risk factor-SNP interactions met our criteria. Namely, two SNPs within the *SLC4A5 *gene (rs828853 and rs12991424) interacted with diabetes, and one SNP within the *ADRB2 *gene (rs1042713) interacted with Lp(a). Region 8 displays the epistatic (SNP-SNP) interactions significantly associated with ABI. We detected 32 replicated and cross-validated, statistically significant pairwise interactions between SNPs. This number was reduced to 25 interactions after controlling for multiple testing with FDR. Approximately half of these interactions involved variants in the solute-carrier genes (7 interactions) and *TGFB3 *gene (5 interactions).

To begin to assess the combined predictive ability of the top SNPs, risk factors, and their interactions, we used multivariable modeling techniques and investigated the percent variation in ABI explained in the full sample and in the independent test samples used in the cross validation (i.e. a more accurate estimate of the predictive ability of these variations for other yet to be sampled individuals in this population of inference) (see Table 4). We found that the two single SNPs that met our criteria explained 0.65 percent of variation (adjusted R^2^) in ABI alone (not adjusting for risk factors) and the top four SNP-SNP interactions explained an additional 4.5% of variation in ABI. The covariates explained 12.5% of the variability in ABI alone while the top SNP-covariate interactions explained an additional 2.25% (adjusted R^2 ^= 15.04). After accounting for risk factors and their interactions with SNPs, the top SNP-SNP interactions explained an additional 1.75%. Combining the top SNPs, risk factors, and their interactions into a model explained 17.85% of variation in ABI in the sample. To assess the predictive ability of these models in new individuals from the same population we used cross-validation methods and estimated the CV R^2 ^(see Methods). The predictive ability of the genetic variations appears to be modest, at best, compared to the covariates.

**Table 4:** Multivariable analysis to assess combined predictive ability of the best SNPs, risk factors, and interactions

**Model**	**Adj R^2^**	**CV R^2^**
Single Replicated and CVD SNPs	0.0065	0.0009
Single SNPs + Top 4 SNP*SNP Interactions	0.0515	0.0066
CVD Covariates	0.1252	0.1040
CVD Covariates + Single SNPs	0.1397	0.1178
CVD Covariates + Single SNPs + 3 SNP*Covariate Interactions	0.1504	0.1118
CVD Covariates + Single SNPs + Top 4 SNP*SNP Interactions	0.1652	0.0877
CVD Covariates + Single SNPs + 3 SNP*Covariate Interactions + Top 4 SNP*SNP Interactions	0.1785	0.0963

CVD, cross-validated; SNPs, Single Nucleotide Polymorphisms

## Discussion

Multiple studies have investigated the association of polymorphisms in candidate genes with essential hypertension and coronary heart disease, but relatively few studies have explored the relationship between specific candidate gene polymorphisms and ABI, a marker of PAD. Our motivating hypothesis was that genetic polymorphisms implicated in risk factors for hypertension and CHD may influence PAD risk by means of common pathophysiological pathways. Therefore, in order to understand the genetic architecture of a complex multifactorial trait such as ABI, larger scale investigations of the polygenic network of genes and their impact on underlying physiological and biochemical correlates need to be examined simultaneously [33]. Out of 112 biological and positional candidate genes, SNPs in 30 different genes were related to inter-individual variation in ABI, a non-invasive measure of PAD, in our study. Six of these genes were also associated with underlying physiological correlates.

Even after adjustment for conventional risk factors and stringent type I error reduction techniques, two of the *NOS3 *SNPs shared significant associations with ABI, suggesting that alterations in *NOS3 *may indeed influence inter-individual variation in ABI. We did genotype the well-known *NOS3 *non-synonymous SNP Asp298Glu (rs1799983), which has been postulated to alter function of *NOS3 *[34], but did not find the SNP to be associated with ABI. These findings are consistent with our previous report of an association between polymorphisms in *NOS3 *and inter-individual variation in ABI [7].

Diabetes is one of the main risk factors for PAD. Several studies have identified genetic variants that increase risk for PAD among type 2 diabetics [35-37]. As such, it is plausible that genetic susceptibility to PAD is modified by diabetes status. In line with this, 2 of the 3 SNP-covariate interactions that passed our stringent criteria involved diabetes as the environmental covariate. While the prevalence of diabetes was low in our sample, our results provide preliminary evidence for a gene-environment interaction, even after adjustment for conventional risk factors. This finding underscores the importance of considering the particular contexts that may potentially modify genetic susceptibility to complex disease.

An interesting finding from our study is that the majority of significant genetic effects were in the form of epistatic interactions. This finding provides further evidence that the genetic susceptibility to complex atherosclerotic diseases is not attributable to the modest effects of a single gene and is likely a result of a combination of alleles in multiple genes [6]. Animal and plant studies have also recently shown an abundance of epistatic interactions, more than had previously been expected [38].

In the clinical setting, ABI is used as a dichotomous variable, with cut off values of ≤ 0.90 or ≤ 0.95 employed to confirm the presence of PAD. We did not analyze ABI as a dichotomous variable as this entailed a substantial loss of statistical power, particularly since the prevalence of an abnormal ABI (defined as ≤ 0.90) was low (6.8%) in our study sample. Despite this, our analyses with ABI as a continuous outcome were warranted as recent studies suggest that, even in the range of 1.0–1.3, lower ABI may be related to PAD risk factors [39]. Furthermore, just as genetic variation influencing BP variation in normotensives has been related to an increased risk of hypertension [40], we expected that genetic variation associated with ABI levels might be related to an increased risk of PAD.

An interesting result from this study is the relatively low level of agreement between results filtered through different methods of reducing false positives – namely, adjustment for multiple testing using FDR < 0.30, internal replication, and four-fold cross-validation. One of the shortcomings of genetic association studies is that they have often failed to replicate and Manly [10] suggests that internal validation, common to good experimental practices, is one way to avoid the publication of false positives. In our study, we used cross-validation methods to significantly reduce the chance of false positives. Cross-validation methods were developed in the late 1970's as a way to incorporate a measure of predictive accuracy (and correspondingly, a measure of prediction error) for an estimated model based on its performance predicting the outcome for independent test cases [12]. During the last decade, cross-validation methods have been used widely for everything from robust variable selection in gene expression array studies [41] to reducing false positives in gene-gene interaction studies [42,43] to evaluating the predictive accuracy of molecular or genetic classifiers of disease before clinical implementation [44]. Cross-validation has become a standard in the field of metabolomic [45], proteomic [46,47], and transcriptomic [48] studies because of its ease of execution and its emphasis on prediction in independent test cases as a method of discriminating between true associations and false associations.

We should note that although it appeared in this study that FDR was more conservative than cross-validation or internal replication, this is not always the case. We have conducted similar analyses in other studies (results not shown) and have found cross-validation to be more conservative than the FDR, leading us to the general conclusion that multiple methods should be employed simultaneously to reduce type I errors for genetic association studies.

Concerns have been raised that population stratification may lead to spurious results in genetic association studies [44]. To address this potential impact, we assessed the presence of population substructure using STRUCTURE [49] and found no evidence of subpopulation clusters in our sample. Wacholder et al. have pointed out that "population stratification does not occur in an ethnically homogeneous population" [50] and the bias that may arise in a population-based study of non-Hispanic Caucasians, as a result of ignoring ethnicity, is likely to be very small [51].

Some limitations of the present study need to be considered. Our approach was based on the premise that susceptibility alleles for common diseases (and related subclinical disease measures such as ABI) are not under strong negative selection, and common variants contribute to common disease traits (i.e. the 'common disease – common variant' hypothesis) [52]. However, the allelic spectrum for genes associated with complex quantitative traits such as ABI is not fully delineated, and it is possible that multiple rare polymorphisms in the biological and positional candidate genes that we studied influence ABI. Due to a lack of power, identifying association with ABI using such alleles would not be possible using the approaches employed in this study. Our inferences may not be generalizable to individuals who are younger, normotensive, or of other ethnicities. Although a priori power calculations indicated that we were adequately powered to detect relatively small SNP effects, insufficient sample sizes (full sample and re-sampled subsets) or random measurement error may have limited our power to detect genotype-phenotype associations. Despite some limitations, our approach illustrates the use of SNPs in candidate genes to construct a more complete picture of the genetic architecture of complex traits such as ABI.

## Conclusion

The genetic architecture of complex multifactorial traits includes common genetic variants with small effects as well as gene-gene and gene-environment interactions. We report that candidate gene SNP main effects, SNP-covariate and SNP-SNP interactions contribute to the inter-individual variation in ABI, a marker of PAD. Our findings underscore the importance of conducting systematic investigations that consider a context-dependent framework for developing a deeper understanding of the multidimensional genetic and environmental factors that contribute to complex diseases.

## Competing interests

The authors declare that they have no competing interests.

## Authors' contributions

SLRK and IJK had the original idea of this article, performed the design, participated in the discussion of the results, and wrote the manuscript. MTG performed the analyses, performed the design, participated in the discussion of the results, and wrote the manuscript. EB and STT participated in the discussion of the results.

## Pre-publication history

The pre-publication history for this paper can be accessed here:



## Supplementary Material

Additional file 1 Summary of Genotyped SNPs in Candidate Genes. Click here for file
