# Supplementary material for: Investigating the complex genetic architecture of ankle-brachial index, a measure of peripheral arterial disease, in non-Hispanic whites
Source: BMC Med Genomics. 2008 May 15;1:16. doi: 10.1186/1755-8794-1-16 (PMC2412898; doi:10.1186/1755-8794-1-16)
Supplement: Additional file 1 — Summary of Genotyped SNPs in Candidate Genes. [file 1755-8794-1-16-S1.pdf]

Supplemental Table 1

Summary of Genotyped SNPs in Candidate Genes

Gene Name	Gene Abbr	Chr. Band	Gene Location Start	Gene Location End	# SNPs	SNPs by rs #
amiloride-sensitive cation channel 4, pituitary	ACCN4	2q35	220087295	220111732	7	rs2385538, rs3770234, rs2276643, rs3755065, rs746233, rs1872858, rs1545970
angiotensin I converting enzyme (peptidyl-dipeptidase A) 2	ACE2	Xp22	15489076	15529058	1	rs4646116
adducin 1 (alpha)	ADD1	4p16.3	2815381	2901585	1	rs4961
adducin 2 (beta)	ADD2	2p14-p13	70742771	70848837	12	rs740387, rs2024458, rs17698193, rs4984, rs2072246, rs3755375, rs4852700, rs2110981, rs1541582, rs3771452, rs2270042, + 1 SNP w/o rs #
adrenomedullin	ADM	11p15.4	10283217	10285499	5	rs4399321, rs4641466, rs5004, rs7924631, rs7944706
adrenergic, alpha-2B-, receptor	ADRA2B	2p13-q13	96142352	96145615	4	rs9333567, rs2229169, rs4907299, rs2252697
adrenergic, beta-1-, receptor	ADRB1	10q24-q26	115793804	115795518	1	rs1801253
*b2-adrenergic receptor	ADRB2	5q31-q32	148186368	148188379	3	rs1042714, rs1800888, rs1042713
Advanced glycosylation end product-specific receptor	AGER	6p21.3	32256723	32260001	1	rs1800684
angiotensinogen (serine (or cysteine) proteinase inhibitor, clade A (alpha-1 antiproteinase, antitrypsin), member 8)	AGT	1q42-q43	228904892	228916564	9	rs5050, rs5051, rs699, rs2493134, rs2493132, rs7079, rs5049, rs5046, rs943580
angiotensin II receptor, type 1	AGTR1	3q21-q25	149898354	149943478	1	rs5186

aldehyde dehydrogenase 5 family, member A1 (succinate-semialdehyde dehydrogenase)	ALDH5A1	6p22.2-p22.3	24603175	24645413	3	rs2760118, rs2760185, rs807516
Alstrom syndrome 1	ALMS1	2p13	73466393	73690554	6	rs11685372, rs1052161, rs2056486, rs13008860, rs2017116, rs1052162
arachidonate 5-lipoxygenase-activating protein	ALOX5AP	13q12	30207668	30236556	2	rs10507391, rs9579646
apolipoprotein E	APOE	19q13.2	50100878	50104489	4	rs449647, rs429358, rs7412, rs405509
ATPase, H+ transporting, lysosomal 56/58kDa, V1 subunit B, isoform 1 (Renal tubular acidosis with deafness)	ATP6V1B1	2p13.1	71016505	71046068	18	rs759218, rs11681642, rs4852732, rs3771385, rs967063, rs2072460, rs2072461, rs2270263, rs1006049, rs1024764, rs1024765, rs2072463, rs2215827, rs2239484, rs2239487, rs2266917, + 2 SNPs w/o rs #s
ancient ubiquitous protein 1	AUP1	2p13	74607284	74610482	1	rs10779958
calpain 10	CAPN10	2q37.3	241174817	241187193	8	rs7571442, rs2975765, rs2975760, rs2975766, rs13007017, rs7607572, rs4676348, rs11556469
calpain 3, (p94)	CAPN3	15q15.1-q21.1	40438989	40491807	1	rs28364538
chloride intracellular channel 1	CLIC1	6p22.1-p21.2	31806338	31812320	1	rs400547
cytidine monophosphate-N-acetylneuraminic acid hydroxylase (CMP-N-acetylneuraminate monooxygenase)	CMAH	6p21.32	25189778	25246032	3	rs1002540, rs302969, rs303006
collagen, type XIX, alpha 1	COL19A1	6q12-q13	70633168	70978878	1	rs1736

C-reactive protein, pentraxin-related	CRP	1q21-q23	157948703	157951003	5	rs3093058, rs3093062, rs1417938, rs2808630, rs3093075
cytochrome b-245, alpha polypeptide	CYBA	16q24	87237198	87244958	1	rs4673
cytochrome P450, family 11, subfamily B, polypeptide 2	CYP11B2	8q21-q22	143988976	143996261	1	rs1799998
dynactin 1 (p150, glued homolog, Drosophila)	DCTN1	2p13	74441789	74461472	1	rs909177
DEK oncogene (DNA binding)	DEK	6p23	18332391	18372750	1	rs3476
DEAQ box polypeptide 1 (RNA-dependent ATPase)	DQX1	2p13.1	74598766	74606826	1	rs6546909
Endothelin receptor type A	EDNRA	4q31.22-q31.23	148621579	148685555	3	rs5343, rs5334, rs5333
endothelin receptor type B	EDNRB	13q22	77367616	77447665	1	rs5351
epoxide hydrolase 2, cytoplasmic	EPHX2	8p21-p12	27404561	27458401	10	rs11996801, rs4149259, rs1042032, rs1042064, rs4149243, rs4149253, + 4 SNPs w/o rs#s
coagulation factor XIII, A1 polypeptide	F13A1	6p25.3-p24.3	6089316	6265901	1	rs5988
fibrinogen, B beta polypeptide	FGB	4q28	155703595	155711686	3	rs1800788, rs4220, rs1044291
hypothetical protein FLJ12788	FLJ12788	2p13.1	74563724	74575192	1	rs6707475
gamma-aminobutyric acid (GABA) B receptor, 1	GABBR1	6p21.31	29677984	29708839	1	rs29230
guanine nucleotide binding protein (G protein), beta polypeptide 3	GNB3	12p13	6819635	6826817	1	rs5442
glypican 6	GPC6	13q32	92677095	93853948	9	rs1012693, rs1323625, rs1359126, rs1409174, rs1409177, rs1535692, rs1535693, rs1886928, rs1924115
G protein-coupled receptor 35	GPR35	2q37.3	241217467	241219342	6	rs12468453, rs3749172, rs2975786, rs12468485, rs2975784

G protein-coupled receptor 55	GPR55	2q37	231480277	231498185	9	rs12467769, rs1992188, rs1992187, rs1992186, rs2396777, rs2969126, rs3749073, rs10498253, rs1344927
protein disulfide isomerase family A, member 3	PDIA3	15q15	41825881	41852095	1	rs # unknown
hemochromatosis	HFE	6p21.3	26195487	26203448	2	rs1799945, rs1800562
5-hydroxytryptamine (serotonin) receptor 1B	HTR1B	6q13	78228667	78229839	2	rs6296, rs6298
5-hydroxytryptamine (serotonin) receptor 2B	HTR2B	2q36.3-q37.1	231681198	231698068	4	rs6437000, rs7607338, rs7580967, rs1549339
intercellular adhesion molecule 1 (CD54), human rhinovirus receptor	ICAM1	19p13.3-p13.2	10242778	10258291	3	rs5491, rs5030352, rs3093032
interleukin 10	IL10	1q31-q32	205007570	205012462	5	rs1800872, rs2222202, rs3024492, rs3024493, rs3024498
Interleukin 17	IL17	6p12	52159143	52163395	1	rs3819025
interleukin 1, beta	IL1B	2q14	113303807	113310827	8	rs1143627, rs1143629, rs3917356, rs3136558, rs1143634, rs2853550, rs3917365, rs3917368
interleukin 6 (interferon, beta 2)	IL6	7p21	22733344	22738141	8	rs2069824, rs2069827, rs2069832, rs2069840, rs2069842, rs2069845, rs2069860, rs2069849
insulin receptor substrate 1	IRS1	2q36	227308181	227372719	7	rs2234931, rs2288586, rs1801277, rs1801278, rs3731596, rs4675094, rs1366757
jun D proto-oncogene	JunD	19p13.2	18251570	18253432	1	rs12461751
kidney associated antigen 1	KAAG1	6p22.1	24465109	24466491	1	rs2274305
potassium voltage-gated channel, Isk-related family, member 4	KCNE4	2q36.3	223625170	223626872	3	rs12621643, rs3795884, +1SNP w/o rs#

potassium inwardly-rectifying channel, subfamily J, member 11	KCNJ11	11p15.1	17363373	17366782	3	rs5210, rs5215, rs5219
potassium inwardly-rectifying channel, subfamily J, member 13	KCNJ13	2q37	233339418	233349519	10	rs1446307, rs2293782, rs1973675, rs737028, rs2293780, rs2293781, rs1446308, rs737027, rs1801251, rs1044480
potassium channel, subfamily K, member 16	KCNK16	6p21.2-p21.1	39390460	39398294	1	rs3734618
potassium channel, subfamily K, member 17	KCNK17	6p21.1	39374762	39390176	1	rs10947803
potassium channel, subfamily K, member 5	KCNK5	6p21	39264725	39305229	1	rs1541816
low density lipoprotein receptor (familial hypercholesterolemia)	LDLR	19p13.3	11061131	11105490	10	rs12983082, rs2738444, rs2738460, rs11669576, rs5925, rs6413504, rs2738465, rs1003723, rs14158, +1 SNP w/o rs#
hypothetical protein BC014602	LOC130951	2p13.1	74638526	74728672	3	rs363698, rs363685, rs2021725
hypothetical locus LOC401237	LOC401237	6p22.3	21772092	22322714	1	rs3406
lymphotoxin alpha (TNF superfamily, member 1)	LTA	6p21.3	2986215	2988221	1	rs1041981
major histocompatibility complex, class I, A	HLA-A	6p21.3	30018310	30021633	1	rs2499
major histocompatibility complex, class II, DO alpha	HLA-DOA	6p21.3	33079937	33085367	1	rs2581
hypothetical protein MGC10955	MGC10955	2p13.1	2787675	2789683	4	rs860455, rs702465, rs702466, rs828843
hypothetical protein MGC22014	MGC22014	2p13.1	74126958	74188812	6	rs13019449, rs13027288, rs7584777, rs7601526, rs10164943, rs828869

matrix metalloproteinase 3 (stromelysin 1, progelatinase)	MMP3	11q22.3	102211737	102219552	5	rs645419, rs522616, rs566125, rs639752, rs683878
matrix metalloproteinase 9 (gelatinase B, 92kDa gelatinase, 92kDa type IV collagenase)	MMP9	20q11.2-q13.1	44070953	44078606	7	rs3918251, rs3918254, rs2250889, rs2274756, rs3918262, rs20544, rs9509
MOB1, Mps One Binder kinase activator-like 1B (yeast)	MOBK1B	2p13.1	74235494	74259491	1	rs828891
MRS2-like, magnesium homeostasis factor (S. cerevisiae)	MRS2L	6p22.3-p22.1	24511131	24533788	3	rs13735, rs2793422, rs3469
methylenetetrahydrofolate dehydrogenase (NADP+ dependent) 2, methenyltetrahydrofolate cyclohydrolase	MTHFD2	2p13.1	74279197	74295930	1	rs10187051
neuromedin U receptor 1	NMUR1	2q37.1	232096114	232103446	6	rs3752763, rs10933376, rs3752762, rs4973442, rs3769986, rs3769987
nitric oxide synthase 2A (inducible, hepatocytes)	NOS2A	17q11.2-q12	23107919	23151682	2	rs1137933, rs2297518
nitric oxide synthase 3 (endothelial cell)	NOS3	7q36	150319079	150342608	14	rs1799983, rs1800783, rs2070744, rs3793342, rs1800780, rs3918186, rs3918188, rs3730305, rs891511, rs891512, rs1808593, rs7830, rs3800787, rs3918226
natriuretic peptide precursor C	NPPC	2q24-qter	232498378	232499203	5	rs11900720, rs5261, rs5262, rs5263, rs5267
nucleoporin 153kDa	NUP153	6p22.3	17723247	17814797	1	rs2274136
one cut domain, family member 1	ONECUT1	15q21.1-q21.2	50836644	50869501	2	rs2456525, rs2440332
opioid receptor, mu 1	OPRM1	6q24-q25	154402135	154481999	1	rs1799971
prostate androgen-regulated transcript 1	PART1	5q12.1	59819516	59822848	1	rs12188950

phosphodiesterase 4D, cAMP-specific (phosphodiesterase E3 dunce homolog, Drosophila)	PDE4D	5q12	58302467	58918032	4	rs152312, rs2910829, rs456009, rs702553
phosphoinositide-3-kinase, regulatory subunit 1 (p85 alpha)	PIK3R1	5q13.1	67558217	67633403	2	rs251401, rs706713
phospholipase C, beta 2	PLCB2	15q15	38367391	38387466	3	rs936211, +2 SNPs w/o rs#s
perilipin	PLIN	15q26	88008602	88023595	3	rs2304795, rs1052700, +1 SNP w/o rs#
protein kinase, cAMP-dependent, regulatory, type II, beta	PRKAR2B	7q22	106472413	106589491	3	rs257376, rs3729877, rs2302453
prolactin	PRL	6p22.2-p21.3	22395458	22405709	2	rs1205960, rs6239
HtrA serine peptidase 2	PRSS25	2p12	74610039	74614191	1	rs715407
retinal degeneration, slow	RDS	6p21.2-p12.3	42772317	42798287	1	rs835
Rhesus blood group-associated glycoprotein	RHAG	6p21.1-p11	49680855	49712511	1	rs2753076
sodium channel, voltage-gated, type VII, alpha	SCN7A	2q21-q23	166970134	167051724	7	rs7570585, rs1406275, rs11899387, rs7570585, rs7565062, rs11888208, +1 SNP w/o rs#
sodium channel, nonvoltage-gated 1 alpha	SCNN1A	12p13	6326275	6354976	1	rs2228576
selectin E (endothelial adhesion molecule 1)	SELE	1q22-q25	167958405	167969803	10	rs5353, rs932307, rs5361, rs1534904, rs1076638, rs5368, rs5356, rs3917434, rs3917436, rs5357
selectin P (granule membrane protein 140kDa, antigen CD62)	SELP	1q22-q25	167824713	167866031	4	rs3917724, rs6131, rs6133, rs6136

solute carrier family 12 (sodium/chloride transporters), member 3	SLC12A3	16q13	55456642	55504850	4	rs13306675, rs5804, rs2010501, rs2304483
solute carrier family 17 (sodium phosphate), member 1	SLC17A1	6p23-p21.3	25891295	25938776	4	rs1165153, rs1165196, rs1165209, rs1747522
solute carrier family 17 (sodium phosphate), member 2	SLC17A2	6p21.3	26020968	26038818	3	rs1540273, rs1865760, rs2071299
solute carrier family 17 (sodium phosphate), member 3	SLC17A3	6p21.3	25953307	25990493	3	rs1165160, rs1165165, rs1780966
solute carrier family 17 (sodium phosphate), member 4	SLC17A4	6p22-p21.3	25862944	25888419	4	rs1892251, rs2275905, rs2275906, rs9295669
solute carrier family 19, member 3	SLC19A3	2q37	228258170	228290989	10	rs932134, rs4973234, rs1819, rs13007334, rs4973216, rs13025803, rs2396470, rs12185721, rs4973225, rs6436729
solute carrier family 20 (phosphate transporter), member 1	SLC20A1	2q11-q14	113119997	113137869	10	rs11123145, rs4849091, rs4849093, rs3827758, rs885346, rs6793, rs885347, rs10758, rs1053652, rs1061254
solute carrier family 22 (extraneuronal monoamine transporter), member 3	SLC22A3	6q26-q27	160689414	160796003	1	rs668871
solute carrier family 2 (facilitated glucose transporter), member 2	SLC2A2	3q26.1-q26.2	172196830	172227462	1	rs5400
solute carrier family 4, anion exchanger, member 3	SLC4A3	2q36	220200947	220214928	5	rs12993807, rs2289782, rs684428, rs2305055, rs635311
solute carrier family 4, sodium bicarbonate cotransporter, member 5	SLC4A5	2p13	74302102	74395658	17	rs13002080, rs828853, rs702462, rs6726450, rs12053233, rs10177833, rs2034454, rs8179526, rs13426653, rs7571842, rs12991424,

						rs3771733, rs828902, rs828863, rs1006502, rs4853018, +1 SNP w/o rs#
solute carrier family 8 (sodium/calcium exchanger), member 1	SLC8A1	2p23-p22	40195648	40510948	1	rs5556
solute carrier family 9 (sodium/hydrogen exchanger), isoform 2	SLC9A2	2q11.2	102602598	102694241	6	rs17833353, rs6708870, rs6756408, rs17027748, rs6757258, +1SNP w/o rs#
serum-response factor	SRF	6p21.1	43246897	43257221	1	rs9395
STAM binding protein	STAMBP	2p13.1	73909654	73943518	5	rs11904306, rs11886071, rs11888468, rs1318987, rs919629
transforming growth factor, beta 3	TGFB3	14q24	75494194	75517242	11	rs3917148, rs3917158, rs2268622, rs3917187, rs3917194, rs3917195, rs3917200, rs3917201, rs2284791, rs3917210, rs3917211
thrombospondin 4	THBS4	5q13	79366746	79414861	1	rs1866389
TRAF and TNF receptor associated protein	TTRAP	6p22.3-p22.1	24758184	24775094	1	rs1129644
vascular cell adhesion molecule 1	VCAM1	1p32-p31	100957884	100977187	15	rs1409419, rs1041163, rs3170794, rs3176860, rs3176861, rs3176862, rs3176867, rs3176869, rs3181088, rs3176874, rs3176876, rs3917016, rs3176878, rs3176879, rs3181092
vascular endothelial growth factor	VEGF	6p12	43845930	43862199	1	rs25648
zinc finger, HIT type 4	ZNHIT4	2p13.1	74535706	74538593	1	rs2268417
Other (not located in genic regions)					2	rs2175, rs2469
Total	112				435	
